# Eye gaze and facial displays of emotion during emotional film clips in remitted patients with bipolar disorder

**DOI:** 10.1192/j.eurpsy.2020.26

**Published:** 2020-02-27

**Authors:** Hanne Lie Kjærstad, Caroline Kamp Jørgensen, Ingrid Broch-Due, Lars Vedel Kessing, Kamilla Miskowiak

**Affiliations:** 1 Copenhagen Affective Disorder Research Centre (CADIC), Psychiatric Center Copenhagen, Copenhagen University Hospital, Rigshospitalet, Denmark; 2 Department of Psychology, University of Copenhagen, Copenhagen, Denmark

**Keywords:** Bipolar disorder, emotion reactivity, film clips, eye-tracking, facial expression

## Abstract

**Background.:**

Aberrant emotional reactivity is a putative endophenotype for bipolar disorder (BD), but the findings of behavioral studies are often negative due to suboptimal sensitivity of the employed paradigms. This study aimed to investigate whether visual gaze patterns and facial displays of emotion during emotional film clips can reveal subtle behavioral abnormalities in remitted BD patients.

**Methods.:**

Thirty-eight BD patients in full or partial remission and 40 healthy controls viewed 7 emotional film clips. These included happy, sad, and neutral scenarios and scenarios involving winning, risk-taking, and thrill-seeking behavior of relevance to the BD phenotype. Eye gaze and facial expressions were recorded during the film clips, and participants rated their emotional reactions after each clip.

**Results.:**

BD patients showed a negative bias in both facial displays of emotion and self-rated emotional responses. Specifically, patients exhibited more fearful facial expressions during all film clips. This was accompanied by less positive self-rated emotions during the winning and happy film clips, and more negative emotions during the risk-taking/thrill-related film clips.

**Conclusions.:**

These findings suggest that BD is associated with trait-related abnormalities in subtle behavioral displays of emotion processing. Future studies comparing patients with BD and unipolar depression are warranted to clarify whether these differences are specific to BD. If so, assessments of visual gaze and facial displays of emotion during emotional film clips may have the potential to be implemented in clinical assessments to aid diagnostic accuracy.

## Introduction

Bipolar disorder (BD) is a chronic psychiatric disorder with recurrences [1], leading to a lower quality of life and an elevated risk of suicide [2,3]. The complexity of the BD phenotype causes high rates of misdiagnosis [4,5] and, consequently, delay in correct diagnosis and adequate treatment [5]. This has led to an immense recent research interest in uncovering endophenotypes (i.e., state-independent intermediate phenotypes) to improve the diagnostic accuracy of BD.

Aberrant emotional reactivity and regulation have been posited as promising endophenotypes for BD [6] that are related to impaired interpersonal and occupational functioning [7–9]. However, extant behavioral studies have provided conflicting findings with respect to which of these emotional cognitive domains are affected and whether such changes are state- or trait-related (for a systematic review on emotional cognition in BD, see [10]). Behavioral paradigms of self-reported emotional states in response to pleasant and aversive stimuli have generally failed to detect significant differences between remitted patients with BD and controls [11–16]. It is plausible that these behavioral measures of emotion processing fail to detect differences between patients with BD and healthy controls (HCs) because of insufficient sensitivity of subjective ratings to detect abnormal brain function. In contrast, functional magnetic resonance imaging (fMRI) studies have consistently found aberrant neural activation in patients with BD during explicit and implicit processing of emotional faces and words during acute mood episodes and in remission [17,18]. However, the high cost of fMRI renders this an unfeasible tool to support diagnostic evaluations in clinical practice. Identification of alternative less costly methods with high sensitivity to abnormal brain responses may therefore represent more feasible tools that can more readably be implemented in the clinical assessments.

Eye-tracking and facial emotion analysis are highly sensitive to emotion processing abnormalities and can more readily be implemented to aid diagnostic accuracy due to their lower costs and easier applications in the clinic. Indeed, eye-tracking studies of facial emotion processing using pro/antisaccade paradigms identified trait-related oculomotor abnormalities, including more errors and slower saccadic responses, in remitted and symptomatic patients [19–21] (although, see also [22], which showed no abnormal viewing patterns in a free-viewing paradigm). Similarly, eye-tracking studies found trait-related negative bias in BD, as reflected by more fixations and time spent viewing threatening images in remitted and symptomatic patients [23,24]. In a recent study, we found that remitted patients with BD exhibited subtle abnormalities in visual gaze patterns and facial displays of emotion when viewing aversive images [16]. Specifically, patients looked more away from these images, which may be an implicit emotion regulation strategy to compensate for their heightened emotional reactivity [16]. In contrast, a study using a free-viewing eye-tracking paradigm showed no aberrant eye-movements in BD [14]. Two studies of facial emotions also found more incongruent facial expressions in remitted patients with BD during presentations of emotional pictures and film clips (i.e., expressions did not match the valence of the emotion-eliciting stimuli) [16,25]. However, one study found no abnormal positive or negative facial displays of emotions during neutral, happy, or sad film-clips compared with controls [26]. The employed film clips in the latter study may have been suboptimal for detection of abnormal emotion processing and regulation in BD. Specifically, film clips tapping into risk-taking and thrill-seeking behavior may be more sensitive to such abnormalities given the clinical presentation of increased impulsivity and risk-taking in BD [27,28]. Also, while static images have limited ecological validity [29], emotional film clips have close resemblance to real-world scenarios and are thus likely to elicit stronger emotional reactivity.

In this exploratory study, we therefore aimed to investigate subtle behavioral differences in emotional reactivity between remitted BD patients and HCs during highly emotional film clips including risk-taking and thrill-seeking scenes using eye-tracking, facial emotion analysis, and self-rated emotional reactivity. Based on the above findings, we hypothesized that patients with BD would: (I) gaze more away from emotional—particularly negative—film clips as an implicit attempt to compensate for their heightened emotional reactivity, (II) display aberrant (e.g., incongruent) facial expressions during all the emotional film clips in general and more facial displays of positive emotions (happiness and surprise) during film clips tapping into risk-taking/thrill-seeking/winning, and (III) experience stronger positive and negative emotional reactions to the pleasant/thrill-related/risk-taking and the aversive/sad/social anxiety-relevant film clips, respectively.

## Materials and Methods

### Participants

Thirty-eight patients with BD and 40 HCs were recruited as part of the larger Bipolar Illness Onset cohort study that aims to identify illness biomarkers in newly diagnosed patients with BD [30]. All participants were screened with the Schedules for Clinical Assessment in Neuropsychiatry to confirm BD diagnosis in patients and ascertain the absence of a psychiatric disorder in HCs, and depressive and manic symptoms were rated with the 17-item Hamilton Depression Scale (HDRS-17) [31] and Young Mania Rating Scale (YMRS) [32]. All patients with BD, 15–70 years of age, from Clinic for Affective Disorders at Psychiatric Centre, Copenhagen were consecutively referred to the study after confirmed ICD-10 diagnosis of BD by their treating psychiatrist. They were included in the study if they were in full or partial remission defined as total scores of ≤14 on the HDRS-17 and YMRS, respectively. Age- and gender-matched HCs were recruited through the Copenhagen University Hospital blood bank or through online advertisement. They were included if they had no personal or first-degree family history of psychiatric illness or substance abuse. Informed consent was obtained from all participants prior to inclusion in the study. Exclusion criteria for all participants were a history of brain injury, neurological disorders including dementia, severe somatic illness, current substance abuse, and having received electroconvulsive therapy in the previous 3 months. Furthermore, participants were excluded if the eye-tracking calibration was not successfully achieved.

### Procedure

Study participation involved a single test session of approximately 2 h at The Psychiatric Centre Copenhagen, Copenhagen University Hospital. During this time, participants were given two experimental tasks: an emotional picture task, for which the results have been reported previously [16] and a paradigm involving emotional film clips. In the latter paradigm, participants were presented with a set of positive and negative emotional film clips on a 14-in. laptop computer screen. Their eye movements and facial expressions were recorded using the Tobii X2-30 eye-tracker (Tobii Inc., Stockholm, Sweden) and laptop camera, and the data were processed using the iMotions software (iMotions Inc., MA, USA). The eye-tracker was initially calibrated using a seven-point calibration, and the participants were instructed to sit comfortably approximately 50 cm from the screen, not to talk, breathe normally, and to keep their eyes on the screen during the entirety of the task.

### Experimental paradigm

Participants were presented with seven emotional film clips; including a *neutral*, a *happy*, and a *sad* film clip. The latter four film clips were specifically chosen to tap into the BD phenotype and involved winning an Olympic medal (*winning*), a thrill-seeking car race (*racing*), walking on top of a skyscraper without safety equipment (*risk-taking*), and a socially uncomfortable situation (*socially anxious*) (see Table 1 for a description of the emotional film clips). The film clips lasted between 90 and 110 s and were presented in a random order. The total duration of the task was 13 min. The task was administered using iMotions software platform version 6.4 on a Lenovo T470s (Beijing, China) laptop computer.

**Table 1.** Narratives of the seven emotional film clips

Film clip	Narrative
Neutral	A man explains how to make a cup of tea while making one himself.
Happy	A mother sits with her four laughing babies. The laughing continues for more than a minute.
Sad	Footage of the Holocaust.
Winning	The Danish swimmer Pernille Blume wins a gold medal at the Olympic Games 2016, while the sports commentators cheer enthusiastically.
Racing	Two cars are racing and at the very last minute, they both avoid crashing into a train.
Risk-taking	A person without safety equipment is walking around and jumping on the roof of a skyscraper.
Socially anxious	A socially awkward situation in which a blindfolded woman farts in front of her friends at her own surprise party, thinking she is alone.

### Measures of emotional reactivity to film stimuli

#### Self-reported positive and negative affect

After viewing each film clip, participants were instructed to rate their level of positive and negative affect, respectively, on a 100-point visual analog scale presented on the screen ranging from *none* (0) to *a lot* (100).

#### Eye-tracking

Total gaze time, fixation time, and fixation count within the area of interest were recoded using the Tobii X2-30 eye-tracker while participants viewed the film clips. This eye-tracking device uses near-infrared light directed at the pupils to generate reflections in the corneas tracked by an infrared camera [33] at a sampling rate of 30 Hz (±2 Hz). The total gaze and fixation times were calculated as the percentage of time participants gazed/fixated within the AOI, while fixation count reflected the number of fixations within the AOI. The AOI was defined as the whole computer screen. Hence, the data reflect how much time is spent actually viewing the film clips rather than looking away. The fixation algorithm was duration dispersion-based, in which gaze points with a 1° radius for a minimum duration of 100 ms with 50% of the samples available is continuously searched for. The fixation centroid (*x*, *y*) was recalculated for each gaze point that was added to the fixation. All data were extracted using the iMotions software.

#### Facial emotion recordings

Participants’ facial expressions were measured using the Affectiva (MA, USA) AFFDEX algorithm. This algorithm detects the face and its action units to outline the facial expression metrics by comparing facial features with a database of normative distributions of feature characteristics [34]. The Affectiva AFFDEX algorithm is based on the Facial Action Coding System (FACS) [35] and uses 14 facial expression metrics to classify the six basic emotions (*joy*, *surprise*, *anger*, *fear*, *sadness*, and *disgust*). We also included a measure of *engagement* (i.e., facial expressiveness) and combined *positive* and *negative* facial expressions (see Table 2 for action units associated with each facial emotion classification). Within the iMotions software platform, the raw data for the six emotions and engagement/expressiveness were calculated to obtain a binary result with a threshold of 10, denoting that facial expressions with at least a 10% likelihood that a human assessor would rate the emotion corresponding to the AFFDEX algorithm were accepted. This threshold was set to detect subtle changes in facial emotions triggered by the film clips and was in accordance with the default settings in the iMotions software. Facial expressions that did each the threshold were considered neutral or a lack of facial expression. All facial expression data were calculated as the percentage of time the facial expression was displayed for each film clip.

**Table 2.** Emotions with corresponding action units and Facial Action Coding System (FACS) descriptions [33,34]

Emotion	Action units	FACS descriptions
Joy	6, 12	Cheek raiser, lip corner puller
Anger	4, 5, 7, 23	Brow lowerer, upper lid raiser, lid tightener, lip tightener
Surprise	1, 2, 5, 26	Inner brow raiser, outer brow raiser, upper lid raiser, jaw drop
Fear	1, 2, 4, 5, 7, 20, 26	Inner brow raiser, outer brow raiser, brow lowerer, upper lid raiser, lid tightener, lip stretcher, jaw drop
Sadness	1, 14, 15	Inner brow raiser, brow lowerer, lip corner depressor
Disgust	9, 15, 16	Nose wrinkler, lip corner depressor, lower lip depressor
Engagement	1, 2, 4, 6, 9, 12, 15, 17, 18, 24, 25, 28	Inner brow raiser, outer brow raiser, brow lowerer, cheek raiser, nose wrinkler, lip corner puller, lip corner depressor, chin raiser, lip puckerer, lip pressor, lips part, lip suck

### Statistical analysis

We conducted a series of repeated-measures analyses of variance with group (BD, HC) as between-subjects factor and emotional reactivity for the seven film clips as within-subjects factor. Statistically significant interactions were followed up with independent samples *t*-tests to examine the origin of the interaction and post hoc analyses of covariance adjusting for depressive and manic symptoms (HDRS-17 and YMRS scores) and years of education. Self-report variables were arcsine-transformed prior to analyses. Finally, we conducted exploratory post hoc correlational analyses between the variables where we found statistically significant group differences and (a) patients’ medication status (yes/no antidepressants, antipsychotics, anticonvulsants, and lithium) and (b) subsyndromal symptoms (HDRS-17 and YMRS scores). Analyses were performed with Statistical Package for Social Sciences (version 22.0, IBM, NY, USA). All effects are reported as significant at *p*s ≤ 0.05 (two-tailed), and effect sizes for significant results are reported as partial eta squared (*ƞ_p_* ^2^) and Cohen’s *d.*

## Results

### Participant characteristics

Six participants (three BD patients and three HCs) were excluded due to incomplete data, resulting in a final sample of 38 patients with BD in full or partial remission and 40 HCs. The two groups were matched for age and sex (*p*s > 0.05). However, patients had undergone fewer years of education (*p* = 0.01) and displayed more subsyndromal depression and mania symptoms (*p*s ≤ 0.001; Table 3).

**Table 3.** Participant demographic characteristics

	Bipolar disorder	Healthy controls	Statistics	df	*p* (two-tailed)
	Mean (SD)	Mean (SD)			
*N*	38	40			
Age	31.3 (9.8)	35.8 (10.4)	*t* = −1.966	76	0.053
Gender (% female)	63	50	*χ* ^2^ = −1.372	1	0.241
Education, years	14.5 (2.8)	16.2 (2.4)	*t* = −2.855	76	0.006
HDRS-17	6.0 (4.2)	1.0 (1.1)	*t* = 7.048	42.30	<0.001
YMRS	3.2 (3.2)	0.7 (1.0)	*t* = 4.557	43.93	<0.001
BD type (% BD-II)	60.5				
Illness duration, years^a^	6.4 (6.5)				
Untreated BD, years^b^	4.7 (6.6)				
Antidepressants, no (%)	4 (10.5)				
Antipsychotic, no (%)	13 (34.2)				
Anticonvulsants, no (%)	15 (39.5)				
Lithium, no (%)	9 (23.7)				

a Defined as time from first manic, hypomanic, or mixed episode to date of test.

b Defined as time from first manic, hypomanic, or mixed episode to date of diagnosis.

### Eye-tracking

Patients with BD generally spent less time gazing at all film clips compared to HCs (main effect of group: *F*(1, 76) = 8.17, *p* = 0.005, *ƞ_p_* ^2^ = 0.097). There was also a significant group by film clip interaction (*F*(6, 456) = 2.32, *p* = 0.03, *ƞ_p_* ^2^ = 0.03); patients with BD spent less time gazing at the *neutral* (*t* = 2.81, df = 60.94, *p* = 0.007, *d* = .64), *happy* (*t* = 2.50, df = 47.13, *p* = 0.02, *d* = .57), *sad* (*t* = 2.31, df = 56.68, *p* = 0.02, *d* = .53), *winning* (*t* = 2.56, df = 65.05, *p* = 0.01, *d* = .58), *racing* (*t* = 2.34, df = 46.13, *p* = 0.02, *d* = .55), *socially anxious* (*t* = 2.62, df = 52.36, *p* = 0.01, *d* = .60), but not *risk-taking* (*p* = 0.83) film clips compared to HCs. These group effects rendered nonsignificant after adjustment for subsyndromal symptoms and years of education (*p*s ≥ 0.12; covarying only for subsyndromal symptoms: *p*s ≥ 0.12). There were no significant differences between the groups in fixation time or numbers of fixations at the film clips (*p*s ≥ 0.50).

### Facial expressions

**Figure 1.**
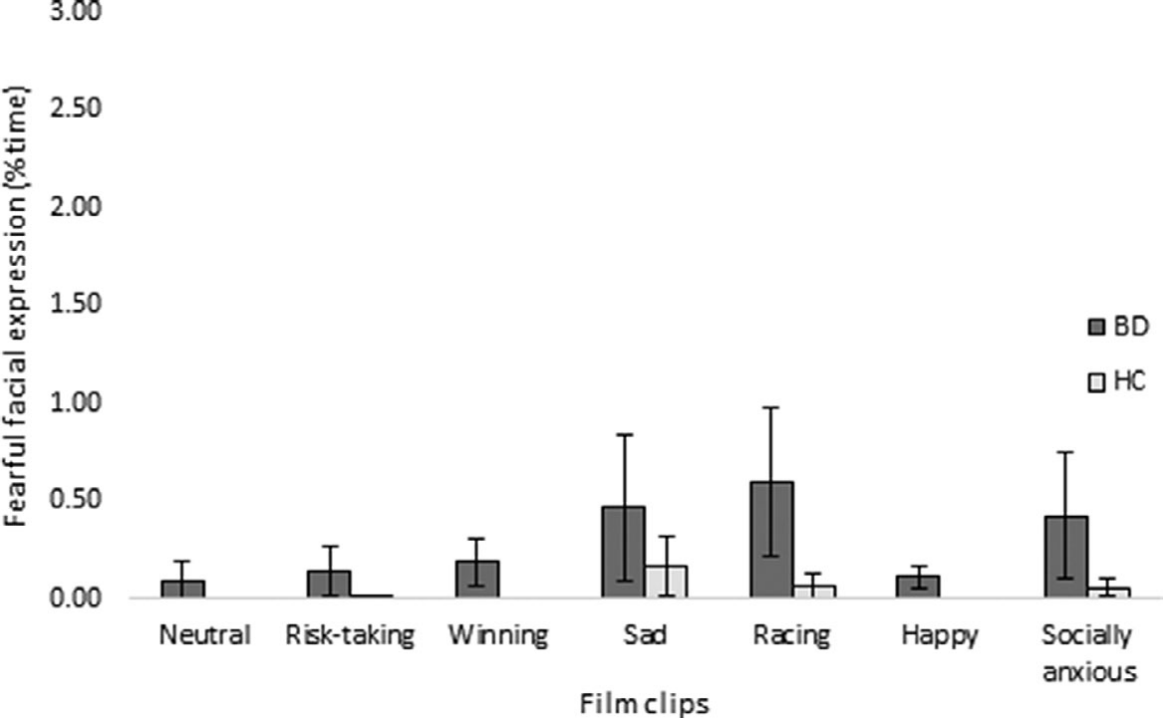
Patients with bipolar disorder (BD) generally exhibited more subtle fearful facial expressions when viewing emotional film clips compared to healthy controls (HCs). Values represent the mean percent fearful facial expressions in patients (BD) and HCs, respectively. Error bars represent standard error of the mean.

Facial expression analyses revealed a trend toward BD patients generally exhibiting more *fearful* facial expressions when viewing film clips than HCs (*F*(1, 75) = 8.13, *p* = 0.08, *ƞ_p_* ^2^ = 0.041), which rendered significant after adjustment for subsyndromal symptoms and years of education (*F*(1, 72) = 15.35, *p* = 0.01, *ƞ_p_* ^2^ = 0.081; Figure 1). This increase in facial displays of fear occurred in the absence of differences between groups in their expressions of joy, surprise, anger, sadness, disgust, or engagement (*p*s ≥ 0.22). Due to technical issues, facial expression data were lost from one BD patient.

### Self-reported positive and negative emotional reactivity

The results revealed a significant group by film clip interaction (*F*(6, 456) = 2.17, *p* = 0.045, *ƞ_p_* ^2^ = 0.03) for self-reported *positive* emotional reactions to the film clips, which prevailed after adjustment for subsyndromal symptoms and years of education (*p* = 0.01). This was driven by less positive emotion ratings by patients with BD for the film clips depicting *winning* (*t* = 2.28, df = 76, *p* = 0.03, *d* = .52) and *racing* (*t* = 2.24, df = 70.15, *p* = 0.03, *d* = .51) and a trend toward less positive emotion ratings in response to the *happy* film clip (*t* = 1.85, df = 76, *p* = 0.07, *d* = .42). There were no significant differences between patients and HCs on self-reported positive reactions to *sad*, *risk-taking*, or *socially anxious* film clips (*p*s ≤ 0.15). However, there was a significant interaction for self-reported *negative* emotional reactivity (*F*(6, 456) = 2.88, *p* = 0.009, *ƞ_p_* ^2^ = 0.04), which was driven by BD patients rating the film clip depicting *risk-taking* as eliciting more negative emotions (*t* = 2.03, df = 76, *p* = 0.046, *d* = .46) compared with HCs. This interaction was reduced to trend-level in a post hoc analysis adjusted for symptom severity and education (*p* = 0.10). No other group differences reached statistical significance (*p*s ≥ 0.12).

### Correlations with medicine and subsyndromal symptoms

No significant correlations were found between medication status (whether patients were taking lithium, anticonvulsants, antidepressants, or antipsychotics) and self-reported emotional reactivity or gaze time (*p*s ≥ 0.08). Patients’ more fearful facial expressions when viewing the film clips depicting *winning* and *racing* was associated with use of anticonvulsants (winning: *r* = 0.34, *p* = 0.04; racing: *r* = 0.33, *p* = 0.045), while more fearful facial expressions when viewing the *socially anxious* film clip was associated with use of antidepressants (*r* = 0.44, *p* = 0.007).

Across all participants, more subsyndromal depressive symptoms were associated with (a) more fearful facial expressions during the *socially anxious* film clip (*r* = 0.24, *p* = 0.036); (b) less gaze time during the *neutral* (*r* = −0.23, *p* = 0.04), *sad* (*r* = −0.23, *p* = 0.04), *racing* (*r* = −0.29, *p* = 0.01), *happy* (*r* = −0.23, *p* = 0.04), and *socially anxious* (*r* = −0.41, *p* < 0.001) film clips; (c) less self-reported positive reactions to *winning* (*r* = −0.23, *p* = 0.04) and *happy* (*r* = −0.28, *p* = 0.01) film clips, and (d) increased self-reported negative reactions to *risk-taking* (*r* = 0.24, *p* = 0.03). Subsyndromal mania symptoms did not correlate with fearful facial expressions, gaze times, or self-reported emotional reactions (*p*s ≥ 0.25).

## Discussion

The present study investigated for the first time eye movements, facial displays of emotion, and self-reported emotional reactivity in response to highly emotional film clips in remitted newly diagnosed BD patients compared with HCs. Consistent with our hypothesis, patients with BD gazed more away during all emotional film clips, although this finding rendered nonsignificant after adjustment for subsyndromal mood symptoms. In contrast, we found no support for our hypothesis that patients would display more extreme positive and negative emotional reactivity for the pleasant/thrill-related/risk-taking film clips and the aversive/sad/social anxiety film clips, respectively. Instead, patients demonstrated a general *negative* bias on both facial emotion recordings and self-reported emotional response, as reflected by stronger facial displays of fear during all emotional film clips combined with less positive emotion ratings in response to the winning and happy film clips and more negative emotion ratings for the risk-taking/thrill-related film clip.

The finding that patients gaze more away from emotional film clips is consistent with our previous finding from an overlapping cohort that patients gazed away from aversive pictures [16]. As participants had been specifically instructed to keep their eyes on the screen even if the scenes played out were unpleasant, this could represent an implicit (nonconscious) emotion regulation strategy in these remitted patients that compensates for their heightened emotional reactivity. Our finding that this difference between BD patients and HCs disappeared after adjustment for subsyndromal depression and mania symptoms, coupled with the significant correlation between eye gaze and subsyndromal depressive symptoms, is also consistent with our previous finding that patients inclination to gaze away from aversive pictures was associated with subsyndromal symptoms [16]. Also, research has suggested that depressed BD patients are less attentive to positive cues, as evidenced by studies showing that depressed BD patients were slower and less accurate at identifying happy words [36], exhibited attentional inference by positive words [37], and spend less time gazing at happy images [24]. Hence, possible implicit attempts to regulate emotional states by controlling gaze direction are likely a state-dependent feature related to subsyndromal symptoms rather than being a trait-related endophenotype of BD.

Few studies to date have investigated subtle behavioral responses to emotional stimuli in remitted patients with BD using facial emotion analysis techniques. Our results showing that patients with BD exhibited subtle aberrant facial expressions during emotional film clips are in accordance with previous studies that have found aberrant facial expressions, specifically increased surprised facial expressions during presentation of neutral and unpleasant images [16] and less appropriate facial expressions when viewing emotional film clips in remitted patients with BD compared to HCs [25]. This is consistent with the results of the present study, in which patients with BD expressed more fear, even during film clips considered positive (i.e., *happy* and *winning*). Interestingly, there was an association between greater use of anticonvulsants and more fearful facial expressions in patients with BD during the *winning* and *racing* film clips involving competition, risk-taking, and thrill-seeking behavior. It is well-established that there is a close association between facial displays of emotion and the emotion that is subsequently experienced at a conscious level [38]. It could therefore be speculated that this may be a neuropsychological mechanism by which anticonvulsants counteract escalation of positive emotion into manic episodes, although this post hoc finding should be interpreted with caution.

Results from previous studies investigating reactivity to emotional stimuli using self-report measures of valence in patients with BD have been inconsistent. In accordance with the results of our study showing no differences between patients and HCs on self-reported emotional reactivity to happy, sad, and neutral film clips, a majority of previous studies report no aberrant self-reported emotion reactivity to unpleasant, pleasant, and neutral images [11,16,39–41]. Other studies, however, have found that remitted patients with BD report greater *positive* emotional responses in response to happy, sad, and neutral film clips and neutral pictures, respectively [42,43]—possibly reflecting a state-dependent feature of mania only observable in samples of BD-I patients. Our study is the first to suggest that film clips associated with mania-like behavior, such as competition and thrill-seeking behavior (i.e., film clips depicting winning and racing) elicited *less positive* or *more negative* emotions in newly diagnosed remitted patients with BD compared to HCs.

Our results suggest that patients with BD demonstrated a trait-related negative bias—both at the overt self-report level and within more subtle measures of facial displays of emotion. This negative bias was evident in our sample of partially/fully remitted patients who were also newly diagnosed with BD, hence further supporting negative bias being trait-related in BD. Indeed, adaptive emotion processing depends on amygdala responses to negative information in healthy populations [44]. Yet hyper-activation of the amygdalae has been associated with trait-related negative bias during processing of neutral and sad faces and emotion regulation in BD (e.g., [17,45,46]). Attentional bias toward negative emotions has also been extensively reported in patients with unipolar depression (UD) and therefore appears not to be specific to BD [47]. In our patient sample, the majority (60.5%) suffered from BD-II, which—given its significant resemblance with UD—may explain the observed negative emotional bias. There is therefore a need for studies to delineate whether the negative bias in emotion reactivity pertains specifically to UD and BD-II patients or to mood disorders in general. Differences in aberrant emotional reactivity in patients with UD and BD-II compared with BD-I may not increase the diagnostic accuracy, which is mostly inappropriate for BD-II, but could help improve treatment selection for BD patients once they have been diagnosed similarly to the recent use of the polarity index [48].

A strength of the present study was that it included a relatively large sample of patients and controls (*N* = 78) compared with previous studies (*N* = 30–46) [16,25,42,49], and that patients were in either full or partial remission. Nevertheless, several limitations should be noted. First, a majority (66%) of the patient sample were taking psychotropic medication at time of testing. The use of antidepressants, antipsychotics, and anticonvulsants have been linked with unfavorable cognitive side-effects, in part because of their anticholinergic and antidopaminergic actions [50,51]. Yet, excluding medicated patients would yield a limited sample size (*N* = 13) as well as possibly reduce the generalizability of the results given the frequent use of psychotropic medication in BD. Second, not all patients were in full remission. However, post hoc sensitivity analyses in patients who were in full remission (defined as HDRS-17 and YMRS ≤7) revealed no changes in the results in comparison to the results of the analyses with adjustment for subsyndromal symptoms and education level. That is, for eye-tracking analyses, results rendered nonsignificant when limiting the sample to patients in full remission (*p* = 0.21). However, for facial expressions, there was a significant effect of group (*F*(1,60) = 4.29, *p* = 0.043), driven by remitted patients with BD exhibiting more fear when watching the film clips. For self-reported emotional reactivity, there was a significant interaction for positive emotions (*F*(4.5, 277.6) = 2.38, *p* = 0.04), driven by remitted patients with BD rating *racing* (*t* = 2.78, df = 60.9, *p* = 0.007) and *winning* (*t* = 2.09, df = 61, *p* = 0.041) as eliciting more negative emotions than HCs. All other effects of group remained nonsignificant (*p*s ≥ 0.10). Third, we did not adjust for multiple comparisons given the exploratory approach of the study. Results are therefore hypothesis-generating in nature. Fourth, the cross-sectional design of the study limits causal inferences. Fifth, a higher sampling rate of the eye-tracker as well as self-ratings of emotions before each emotional film clip would have given more detailed and precise results. Sixth, baseline self-reported emotions ratings conducted prior to the administration of the emotional film clips would have elucidated whether the results were attributable to the emotional film clips or reflected a stable emotion. Finally, given that the negativity bias found in patients with BD in the current study is typically considered a feature of UD, and that the most common misdiagnosis of BD is UD [52], an additional limitation of this study is the lack of a UD comparison group. Future studies should investigate subtle behavioral responses during emotion reactivity between remitted patients with BD and UD versus HCs in order to elucidate whether our findings are BD-specific or a general feature of mood disorders.

## Conclusions

In conclusion, the present study found subtle behavioral differences in facial expressions and self-report measures of emotions in response to emotional film clips between newly diagnosed remitted patients with BD and HCs. Specifically, patients with BD displayed a trait-related *negative* bias in facial expressions and self-rated emotional reactivity to happy/thrill-related/risk-taking film clips. The present preliminary findings suggest that inclusion of highly sensitive behavioral measures like eye-tracking and facial emotion analysis could have the potential to improve clinical decision making on diagnostic accuracy in the clinical settings. Future studies should therefore compare patients with BD to patients with UD to investigate whether these subtle differences are specific to BD or to mood disorders in general.
